# Supplementary material for: Nanosafety: An Evolving Concept to Bring the Safest Possible Nanomaterials to Society and Environment
Source: Nanomaterials (Basel). 2022 May 25;12(11):1810. doi: 10.3390/nano12111810 (PMC9181910; doi:10.3390/nano12111810)
Supplement: Supplementary file 1 [file nanomaterials-12-01810-s001.zip › nanomaterials-1728934-supplementary.pdf]

nanomaterials

Review

Nanosafety: an evolving concept to bring the safest possible nanomaterials to society and environment

Filipa Lebre [1], Nivedita Chatterjee [1], Samantha Costa [2], Eli Fernández-de-Gortari [1], Carla Lopes [1], João Meneses [1], Luís Ortiz [3], Ana R. Ribeiro [1], Vânia Vilas-Boas [1], Ernesto Alfaro-Moreno [1,*]

Supporting Information

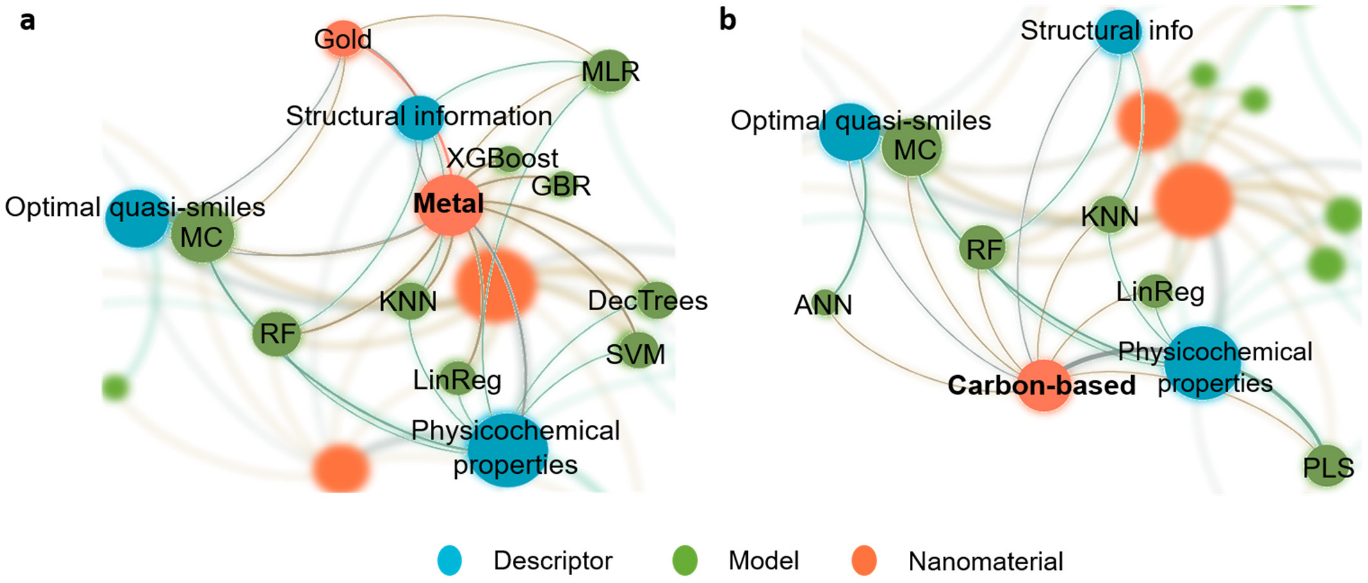

Figure S1. Graphical network of the 19 articles that meet the criteria to be included in Table 5. Most relevant connections of (**a**) metal and (**b**) carbon-based class. ANN: artificial neural networks; DecTrees: decision trees; GBR: gradient boosting regressor; KNN: k-nearest neighbors; LinReg: linear regression; MC: Monte Carlo; MLR: multiple linear regression; PLS: partial least squares; RF: Random Forest; SVM: support vector machine; XGBoost: extreme gradient boosting
